# Impacts of Interleukin-18 Polymorphisms on the Incidence of Delayed-Onset Cytomegalovirus Infection in a Cohort of Kidney Transplant Recipients

**DOI:** 10.1093/ofid/ofz325

**Published:** 2019-07-20

**Authors:** Isabel Pérez-Flores, Jose Luis Santiago, Cristina Fernández-Pérez, Elena Urcelay, María Ángeles Moreno de la Higuera, Natividad Calvo Romero, Beatriz Rodríguez Cubillo, Ana Isabel Sánchez-Fructuoso

**Affiliations:** 1 Nephrology Department, Hospital Clínico San Carlos, Universidad Complutense de Madrid, Madrid, Spain; 2 Kidney Transplant Group Hospital Clínico San Carlos, Madrid, Spain; 3 Clinical Research and Methodology Unit, Hospital Clínico San Carlos, Instituto de Investigación Sanitaria San Carlos, Madrid, Spain

**Keywords:** cohort study, delayed-onset cytomegalovirus infection, IL-18 polymorphism, kidney transplant

## Abstract

**Background:**

The incidence of cytomegalovirus (CMV) infection in solid organ transplant recipients may be reduced by antiviral prophylaxis, but this strategy may lead to delayed-onset CMV infection. The proinflammatory cytokine interleukin (IL)-18 plays a major role in viral host defense responses. This study examines the impacts of 2 single-nucleotide polymorphisms (SNPs) in the promoter region of the *IL-18* gene, -607C/A (rs1946518) and -137G/C (rs187238), on the incidence of delayed-onset CMV infection in patients undergoing kidney transplant.

**Methods:**

This retrospective study analyzed 2 *IL-18* SNPs in consecutive adult kidney transplant recipients using real-time polymerase chain reaction with TaqMan probes. Participants were enrolled over the period 2005–2013 and stratified according to their *IL-18* SNP genotype. The concordance index (Harrell’s c-index) was used as a measure of the discriminatory power of the predictive models constructed with bootstrapping to correct for optimistic bias.

**Results:**

Seven hundred nine patients received transplants in the study period, and 498 met selection criteria. Cytomegalovirus infection and disease incidence were 38% and 7.5%, respectively. In multivariate competing risk regression models, carriers of the -607C/-137G haplotype who received prophylaxis showed a higher incidence of CMV replication after antiviral agent discontinuation (hazard ratio = 2.42 [95% confidence interval, 1.11–5.26]; *P* = .026), whereas CMV disease was not observed in those given prophylaxis who were noncarriers of this polymorphism (*P* = .009).

**Conclusions:**

Our findings suggest that the -607C/-137G *IL-18* haplotype is associated with a higher incidence of postprophylaxis CMV replication. The prior identification of this polymorphism could help select alternative measures to prevent delayed-onset CMV infection in these patients.

Patients undergoing solid organ transplant (SOT) show a high risk of cytomegalovirus (CMV) infection or disease. Because of drug-induced immunosuppression, more than 50% of SOT recipients develop CMV infection during the posttransplant period. Furthermore, if no antiviral prophylaxis or preemptive therapy is provided, up to 10%–50% may develop symptomatic disease [1]. The direct effects of CMV are a viral syndrome or tissue-invasive disease [2]. Possible indirect effects are opportunistic infection [3, 4], acute or chronic graft rejection [5, 6], graft loss [7], and reduced recipient survival [8].

The incidence of CMV disease after SOT may be reduced by antiviral prophylaxis or preemptive therapy. However, antiviral prophylaxis may lead to delayed-onset CMV infection or disease, particularly in CMV-seronegative recipients of organs from CMV-seropositive donors (CMV D^+^/R^−^). After kidney transplantation, both early-onset and delayed-onset tissue-invasive CMV disease have been significantly associated with allograft loss and mortality [9–12].

Some authors report a reduction in delayed-onset CMV disease when antiviral prophylaxis is extended to 6 months posttransplant [13]. However, long-term prophylaxis may inhibit the development of CMV-specific T-cell immunity, because complete viral suppression prevents the host from mounting a CMV-specific immune response. This persistent lack of immunity puts CMV D^+^/R^–^ SOT patients at a high risk of CMV disease soon after antiviral prophylaxis is discontinued [14, 15]. In theory, this high risk of CMV disease is independent of the duration of prophylaxis.

The costs and toxicity of antiviral prophylaxis also remain a concern. Thus, antiviral drugs should be targeted only at kidney transplant patients at a high risk of developing CMV-related complications [16]. To identify this subset of patients, specific kidney transplant risk factors for delayed-onset CMV remain to be defined.

The role played by innate immunity in CMV infection control is not yet fully understood. Studies examining single-nucleotide polymorphisms (SNPs) in genes related to innate immunity have suggested links between certain SNPs, such as those in interleukin (*IL*)*-28B*, Toll-like receptors (*TLR*)*-*9, and interferon lambda (*INFL*)*-3/4*, and the risk of CMV reactivation [17, 18]. However, the potential relationship between CMV and SNPs in *IL-18* gene has not been studied. This pleiotropic proinflammatory cytokine is an important regulator of both innate and acquired immune responses and plays a critical role in inducing T-lymphocyte responses [19]. *IL-18* is mainly produced during the acute immune response by monocytes, macrophages, and immature dendritic cells and participates in cellular and humoral responses. Depending on the immunological context, *IL-18* is involved in both T helper (Th)1 and Th2 immune responses. Because of these multiple functions, *IL-18* is thought to play a major role in host defense against viral infection while, in parallel, the cytokine also induces autoimmune diseases and propagates inflammatory processes [20, 21]. In fact, serum levels of this cytokine have been found elevated during primary CMV infection [22].

The *IL-18* gene has been localized on chromosome 11q22.2–22.3. The SNPs -607C/A (rs1946518) and -137G/C (rs187238) affecting the *IL-18* gene promoter region cause the differential expression of IL-18. Accordingly, the presence of -607C and -137G alleles in the IL-18 promoter promotes the binding of many transcription factors, increasing *IL-18* messenger ribonucleic acid levels [23–25]. Thus, the present study was designed to examine the relationship between both of these SNPs and CMV infection in a cohort of patients undergoing primary kidney transplantation.

## METHODS

### Study Population and Design

Between January 1, 2005 and December 31, 2013, 709 adult patients (≥18 years) consecutively received a deceased donor kidney graft. These patients were under follow-up at our center for at least 24 months. Exclusion criteria for this study were as follows: previous transplant, Caucasian ancestry, graft lost due to primary nonfunction, or death in the immediate postoperative period. These criteria left a final study population of 498 patients (see Supplementary Figure 1).

The study was carried out in accordance with good clinical practice guidelines and the tenets of the Declaration of Helsinki (Medical Research Council 1998) considering ethical principles for human research. The study protocol was approved by our hospital’s review board. Participating patients were informed in detail of the study objectives before providing signed consent.

We also determined the frequency of SNPs in blood samples from a group of healthy subjects from our hospital’s donor blood bank as representative *IL-18* SNP genotypes for the local population. These data were used to confirm expected frequencies according to Hardy-Weinberg equilibrium (HWE) so that we could exclude any polymorphism deviating from this equilibrium because it would not be a good genetic marker for our study.

### Definitions of Cytomegalovirus Infection and Disease

Cytomegalovirus infection was defined as CMV detected in blood in the absence of symptoms. Cytomegalovirus disease was classified according to published guidelines either as CMV syndrome or tissue-invasive disease [26]. Cytomegalovirus syndrome was defined as CMV detected in blood with at least 2 of the following: fever ≥38ºC, malaise, leukopenia or neutropenia, ≥5% atypical lymphocytes, thrombocytopenia, elevation of hepatic aminotransferases to 2 times the upper limit of normal. In contrast, tissue-invasive CMV disease was diagnosed when there were clinical symptoms and signs of tissue invasion accompanied by detection of CMV in a blood specimen and virologic and/or histologic detection of CMV in a biopsy specimen.

### Cytomegalovirus Prophylaxis and Diagnostics Methods

Over the study period, CMV was detected in blood samples using 2 methods: pp65 antigenemia until the year 2011, and Argene (bioMérieux) real-time CMV polymerase chain reaction (PCR) in plasma (with lower limit of quantitation of 150 copies/mL) from 2011 onwards. Each patient was monitored for CMV replication every week during the first posttransplant month, every 2–3 weeks until 6 months, and every 6–8 weeks from 6 to 12 months. Antiviral therapy was given to asymptomatic patients showing CMV replication indicated by the presence of antigens in blood or by a CMV deoxyribonucleic acid level equal to or greater than 400–600 copies/mL of plasma.

According to the protocol of our center, prophylaxis is given to all CMV D^+^/R^–^ patients and to all those receiving lymphocyte-depleting antibodies regardless of their CMV serostatus. Antiviral prophylaxis is started within the first 1–2 weeks of transplant and maintained for 3 or 6 months (3 months until 2009 and 6 months after this date) in CMV D^+^/R^–^ patients and for 3 months in seropositive recipients under lymphocyte-depleting immunosuppression. The antiviral agent used was ganciclovir or valganciclovir depending on whether the estimated glomerular filtration rate was lower or higher than 15 mL/minute, respectively, adjusting dose for renal function.

### Deoxyribonucleic Acid Extraction and Genotyping of the *IL-18* Promoter Region

Deoxyribonucleic acid was isolated from blood samples collected into ethylenediaminetetraacetic acid-coated tubes by the salting out procedure or by automatic methods (MagNA Pure) and quantified, and its purity was determined in a Nanodrop spectrophotometer. The SNPs -607C/A (rs1946518) and -137G/C (rs187238) were analyzed by conducting TaqMan assays in a 7900HT real-time PCR system (Applied Biosystems, Foster City, CA).

### Statistical Analysis

Recipient, donor, and transplant characteristics are summarized using descriptive statistics. Quantitative variables are expressed as the mean and standard deviation or the median and interquartile range. Qualitative variables are provided as frequency distributions.

The χ ^2^ or Fisher’s exact test was used to assess relationships between qualitative variables when more than 25% of expected frequencies were less than 5. The behavior of quantitative variables was assessed for each of the independent variables categorized by the Student’s *t* test (in comparisons of 1 variable with 2 categories). In the case of asymmetry, nonparametric tests such as the median test were used. Pairwise linkage disequilibrium was tested with the HapMap, and deviation from the HWE for each SNP was determined in a χ ^2^ test.

Cumulative probabilities of developing CMV infection and/or disease were graphically examined using Kaplan-Meier survival curves, and differences between haplotypes were tested using the log-rank statistic. A multivariable Cox proportional hazards model was used to analyze potential risk factors for CMV infection and/or disease, with results expressed as hazard ratios (HRs) with 95% confidence intervals (CIs). This prediction model was constructed according to the TRIPOD statement and variables included when *P* < .15 in the univariate analysis or if they emerged as biologically relevant in the population analysis. Interactions were examined between antiviral prophylaxis and *IL-18* promotor SNP haplotype. The *P* value for these interactions was obtained from the models constructed. Discriminatory power was assessed using Harrell’s c-index and the study was internally validated using bootstrap methods. The optimistic bias was also calculated. All statistical tests were performed using the software package SPSS version 21.0 and STATA version 15.0.

## RESULTS

The 498 kidney transplant patients included in this study were of mean age 53; 66% were men. The incidences of CMV infection and disease in the kidney transplant recipients were 38% and 7.5%, respectively. Fifty-five percent of the recipients (n = 273) had received antiviral prophylaxis. Induction therapy was used in more than two thirds of patients: 40% received thymoglobulin and 37% monoclonal antibodies directed against the alpha chain of IL-2 receptor, basiliximab, or daclizumab. The maintenance immunosuppressive regimen included an antimetabolite, corticosteroids, and calcineurin inhibitors, except in 18 patients (6%) who received a mammalian target of rapamycin (mTOR) inhibitor combined with calcineurin inhibitors or mycophenolate. Cumulative incidences of CMV infection and disease were 0.37 and 0.09 at 12 months posttransplant, respectively. The incidence of CMV replication did not reach statistical significance according to the use of antiviral prophylaxis: 35% (95 of 271) in patients not receiving CMV prophylaxis and 43.7% (93 of 213) in those given prophylaxis (*P* = .060). The median time of viral replication was delayed in patients who received prophylaxis compared with those given preemptive therapy: 4.8 (2.6–6.7) vs 1.4 (1.0–2.5) months after transplant. In patients undergoing prophylaxis, CMV infection was detected 1 (0.5–2.4) month after antiviral agent discontinuation. Only 1 patient acquired CMV infection during prophylaxis, which was resolved by increasing the antiviral agent dose. No viral mutations were detected.

The baseline characteristics of the kidney transplant patients by *IL-18* promotor SNP haplotype (-607C/-137G carrier or CG noncarrier) and main clinical outcomes are provided in Table 1. There were no significant differences in demographic or clinical characteristics between the 2 groups. Nevertheless, there was a higher proportion of patients treated with mTOR inhibitor in the CG noncarrier group.

**Table 1.** Baseline Characteristics and Posttransplant Events of Patients by *IL-18* Gene Promoter Polymorphism Haplotype

IL18 Haplotypes	-607C/-137G Carriers (n = 419) n (%)	-607C/-137G Noncarriers (n = 79) n (%)	*P*
Donor age, years^a^	43.7 (13.6)	43.7 (15.2)	.980
Recipient age, years^a^	52.2 (13.4)	52.6 (12.1)	.825
Donor sex, male	301 (72)	60 (76)	.445
Recipient sex, male	282 (67)	48 (60)	.247
Cold ischemia time, hours^b^	18.1 (16.2–21.1)	18.0 (15.5–20.7)	.417
Time on dialysis, months^b^	16.7 (5.8–30.9)	17.2 (7.0–27.0)	.672
Thymoglobulin treatment	159 (38)	38 (48)	.116
imTOR treatment	13 (3)	6 (8)	.016
CMV serostatus (D^+^/R^−^)	64 (15)	8 (10)	.273
CMV prophylaxis	230 (55)	47 (59)	.527
Sensitized patients	28 (7)	3 (4)	.403
HLA mismatch^a^	4.2 (1.1)	4.3 (1.2)	.237
Delayed graft function	201 (48)	33 (42)	.413
Acute rejection	71 (17)	16 (21)	.511
CMV infection	167 (40)	18 (23)	.013
CMV disease	33 (8)	0 (0)	.009
Loss graft	29 (7)	9 (12)	.096
Exitus	25 (6)	4 (5)	.817
Follow-up, months^b^	59.1 (34.9–89.0)	55.1 (32.8–88.1)	.743

Abbreviations: CMV, cytomegalovirus; HLA, human leukocyte antigen; IL, interleukin; imTOR, inhibitors of mammalian target of rapamycin; IQR, interquartile range.

^a^Data expressed as mean (standard deviation).

^b^Data expressed as median (IQR).

*IL-18* promotor SNP genotype frequencies for the overall cohort were as follows: for rs1946518, 32.1% (n = 146) was CC, 51.9% (n = 236) CA, and 16% (n = 73) AA; for rs187238, 49.9% (n = 227) was GG, 43.5% (n = 198) GC, and 6.6% (n = 30) CC. Genotype data did not significantly deviate from the expected frequencies according to HWE. Finally, the IL-18 haplotype distribution was as follows: 31.4% (n = 228) for AC, 16.1% (n = 117) for AG, and 52.5% (n = 382) for CG.

**Figure 1.**
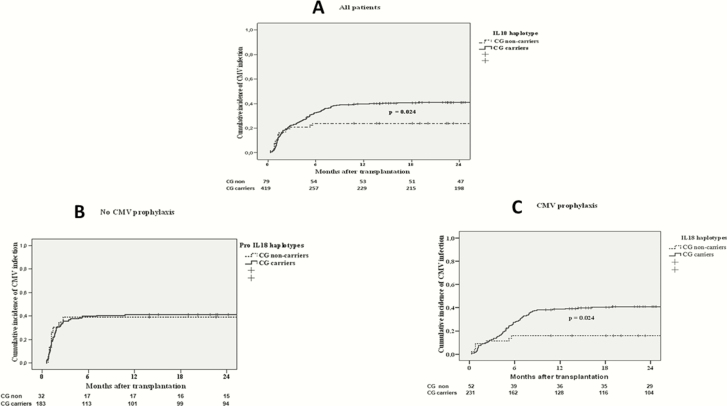
Cumulative incidence of cytomegalovirus (CMV) infection in (A) all patients, (B) patients not receiving CMV prophylaxis, and (C) patients receiving CMV prophylaxis by *IL-18* polymorphism haplotype.

**Figure 2.**
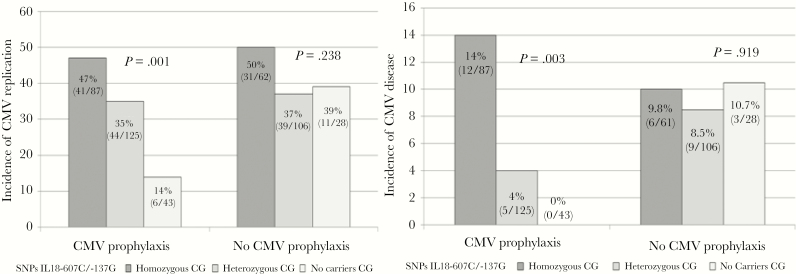
Incidence of cytomegalovirus (CMV) infection and disease by *IL-18* polymorphism haplotype.

In the univariate analysis, the factors associated with an increased risk of CMV infection were as follows: recipient age, cold ischemia time, delayed graft function, CMV prophylaxis, thymoglobulin, mTOR inhibitor, vascular rejection, and CG carriers of the *IL-18* haplotype (Table 2). The duration of prophylaxis did not affect the results of our study (data not shown). The 12-month cumulative incidence of CMV replication was higher among CG polymorphism carriers than the remaining patients (0.41 vs 0.23, *P* = .024) (Figure 1A). Furthermore, an interaction was observed between *IL-18* haplotype and CMV prophylaxis. The risk of CMV infection and/or disease was no different according to *IL-18* haplotype in patients who did not receive prophylaxis (Figure 1B). However, among those given prophylaxis, the cumulative incidence of CMV replication was higher in CG carriers than in CG noncarriers (0.38 vs 0.17, *P* = .024) (Figure 1C). In addition, the risk of CMV infection and disease increased progressively in heterozygous and homozygous CG carriers, and the lowest incidences were detected in CG noncarriers (14% CMV infection, 0% CMV disease) (Figure 2). This effect remained significant in the multivariate model after adjustment for other risk factors associated with CMV replication such that patients with the CG haplotype had a greater risk of postprophylaxis CMV infection than those not carrying this polymorphism (HR = 2.42 [95% CI, 1.11–5.26]; *P* = .026) (Table 3]. The discriminatory power of the model was 0.67 and the optimistic bias 0.03. Due to the existence of 2 periods with different tests for the detection of CMV, a sensitivity analysis was carried out to compare the key results for the general population of the study with patients of the contemporary era. The relationship between postprophylaxis CMV replication and *IL-18* polymorphism is very similar in patients who received transplants as of 2011 (HR = 2.58 [95% CI, 0.90–7.38]; *P* = .076); obviously, the power of test is decrease because there are lower number of subjects.

**Table 2.** Univariate Analysis of CMV Infection

Variable	1-Year Cumulative Incidence of CMV Infection (SE)	HR (95% CI)	*P* Value
Recipient Age			.013
<60 years	33 (2.6)	1	
≥60 years	44 (3.9)	1.45 (1.08–1.96)	
Recipient Sex			.988
Male	37 (2.7)	1.00 (0.73–1.36)	
Female	37 (3.7)	1	
Donor Age			.428
<60 years	36 (2.3)	0.81 (0.49–1.34)	
≥60 years	44 (7.9)	1	
Time on Dialysis			.188
≤24 months	39 (2.7)	1	
>24 months	32 (3.6)	0.80 (0.59–1.10)	
Cold Ischemia Time			.041
≤18 hours	32 (3.1)	1	
>18 hours	42 (3.1)	1.36 (1.01–1.83)	
Delayed Graft Function			.012
Yes	42 (3.2)	1.46 (1.08–1.96)	
No	32 (2.9)	1	
CMV Prophylaxis			.017
Yes	33 (2.9)	1	
No	41 (3.4)	1.38 (1.03–1.86)	
CMV Serostatus			.594
D^+^/R^−^	42 (5.9)	1	
R^+^	36 (2.4)	0.89 (0.60–1.33)	
Thymoglobulin Treatment			.007
Yes	31 (3.3)	0.65 (0.48–0.89)	
No	40 (2.9)	1	
Immunosuppressive Treatment			.032
Tacrolimus	38 (2.2)	1	
mTOR inhibitor	6 (5.4)	0.116 (0.01–0.82)	
HLA Mismatch			.143
≤3	16 (1.0)	1	
>3	37 (2.2)	2.83 (0.70–11.43)	
Acute Rejection			<.001
Yes	51 (5.2)	1.98 (1.42–2.75)	
No	33 (2.4)	1	
IL-18 -607 A/C Polymorphism			.043
CA/CC	38 (2.5)	1.68 (1.01–2.77)	
AA	24 (5.1)	1	
IL-18 -137 C/G Polymorphism			.059
CG/GG	37 (2.4)	2.36 (0.96–5.75)	
CC	17 (7.0)	1	
IL-18 Haplotype			.043
CG carrier	38 (2.5)	1.68 (1.01–2.77)	
CG noncarrier	24 (5.1)	1	

Abbreviations: CI, confidence interval; CMV, cytomegalovirus; HLA, human leukocyte antigen; HR, hazards ratio; IL, interleukin; mTOR, mammalian target of rapamycin; SE, standard error.

**Table 3.** Multivariate Analysis of CMV Infection

Variable	^a^HR (95% CI)	*P* Value
Recipient Age		.010
<60 years	1	
≥60 years	1.51 (1.10–2.08)	
Acute Rejection		<.001
Yes	2.11 (1.49–2.99)	
No	1	
Delayed Graft Function		.048
Yes	1.39 (1.01–1.93)	
No	1	
CMV Prophylaxis		.026
CG carrier	2.42 (1.11–5.26)	
CG noncarrier	1	
No CMV prophylaxis	0.86 (0.44–1.68)	.661
CG carrier	1	
CG noncarrier	-	
Cold Ischemia Time		.035
≤18 hours	1	
>18 hours	1.40 (1.02–1.93)	

Abbreviations: CI, confidence interval; CMV, cytomegalovirus; HLA, human leukocyte antigen; HR, hazards ratio.

^a^Variables included in the model: thymoglobulin treatment, maintenance immunosuppressive therapy, HLA mismatch, CMV serostatus, delayed graft function. *P* interaction (CMV prophylaxis and CG haplotype) = .044.

## DISCUSSION

This study examines the impacts of several recipient, donor, and transplant characteristics on delayed-onset CMV infection and/or disease in a cohort of kidney transplant patients. Although it is possible to reduce the incidence of delayed-onset CMV by extending antiviral prophylaxis [27], in our cohort cases continued to arise after prophylaxis such that strategies for preventing delayed-onset CMV are warranted. One such strategy would be to identify patients at a particularly high risk of infection based on knowledge of its predictive factors. In this study, we observed that the *IL-18* promotor SNP haplotype had a strong influence on the incidence of CMV infection and/or disease, but only among the patients who received prophylaxis. Thus, being a carrier of the -607C/-137G polymorphism emerged as an independent risk factor for CMV replication after stopping prophylaxis. In contrast, the -607A/-137C haplotype conferred a protective effect against CMV replication. This finding points to a critical role of IL-18 in triggering CMV replication after prophylaxis. Unexpectedly, subjects with the CG haplotype showed a greater risk of viral replication after antiviral therapy. Giedraitis et al [24] found that haplotypes CG and AG showed higher transcription activity than haplotype AC, and -607A has been associated with lower serum IL-18 levels than -607C [23]. Moreover, in this last study, monocytes extracted from -137C carriers produced lower amounts of IL-18 than monocytes obtained from -137G carriers.

The *IL-18* gene promoter SNPs examined here have not been previously related to CMV replication. Accordingly, the mechanisms whereby they could influence susceptibility to CMV infection in kidney recipients receiving or not receiving antiviral prophylaxis have not been established. Nevertheless, several studies have identified close relationships between an individual’s *IL-18* haplotype and hepatitis C virus (HCV) and hepatitis B virus clearance, cirrhosis development, and response to treatment [28–31]. As in our cohort, previous studies found that alleles related to lower IL-18 expression levels were correlated with greater HCV clearance, suggesting that high IL-18 levels promote persistent HCV infection. Consistently, other authors have related the CG haplotype to an increased susceptibility to human immunodeficiency virus (HIV)-1 and human T-lymphotropic virus-1 infection [32, 33] and to a greater risk of lipodystrophy in HIV-1 infection [34].

Interleukin-18 is structurally similar to IL-1β and is a member of the IL-1 superfamily of cytokines. Protein complexes called inflammasomes autocatalytically activate intracellular caspase-1, which cleaves the inactive precursors of IL-1β and IL-18 into bioactive cytokines. The main functions of IL-18 are mediated through the induction of IFN-γ secretion from Th1 cells. Acting synergistically with IL-12, IL-18 leads to Th1 differentiation and is thus important in host defense mechanisms against intracellular bacteria, viruses, and fungi. Recent evidence of the involvement of IL-18 in Th2 differentiation and ultimately immunoglobulin E production from B cells has provided insight into the dual effects of IL-18 on Th1 and Th2 inflammatory responses [21]. The biological activity of IL-18 also plays a role in Th17 cell responses. Recent studies have revealed an important role of Th17 responses in host defense against infection. In bacterial and fungal infections, Th17 responses mediate protective mucosal host defense mechanisms and trigger the release of antimicrobial peptides and chemokines for neutrophil recruitment [35]. However, in viral and parasite infections, the role of Th17 cell responses is not as clear. These responses can inhibit the apoptosis of virus-infected cells and might contribute to persistence of the virus [36]. Hence, the chronic induction of Th17 responses, triggered by pathogens such as viruses that have not been sufficiently cleared, could lead to delayed infection and could in some measure explain our results.

Our study has several limitations. The first was the use of different CMV infection detection methods over the 2 eras (pp65 antigenemia and real-time PCR). Second, we are unable to explain why the SNPs examined had such a clear effect only in the patients who received antiviral prophylaxis. Finally, this was a single-center study and although internal validation was conducted following Transparent Reporting of a multivariable prediction model for Individual Prognosis Or Diagnosis (TRIPOD) recommendations, external validation would be needed.

## CONCLUSIONS

To conclude, these results could be relevant and have translational implications in the optimization of the treatment of patients undergoing SOT. The findings suggest that IL-18 promotor SNPs could be useful predictive markers to optimize prophylaxis against CMV infection in kidney transplant recipients, although large, controlled, multicentric studies are needed to confirm them.

Based on the observation that mTOR inhibitors reduce the incidence of CMV after organ transplantation [37], we proposed that, rather than prolonging prophylaxis, these immunosuppressant drugs be used in carriers of the CG haplotype. It has been described that sirolimus acts selectively on naive T cells and on human memory and improves the function of CMV-specific T cells through the modulation of the environmental environment [38]. In addition, long-term prophylaxis may inhibit the development of CMV-specific T-cell immunity and has toxicity and high costs, as mentioned above.

## Supplementary Data

Supplementary materials are available at Open Forum Infectious Diseases online. Consisting of data provided by the authors to benefit the reader, the posted materials are not copyedited and are the sole responsibility of the authors, so questions or comments should be addressed to the corresponding author.

ofz325_suppl_supplementary_figure_s1
