# Supplementary figures and images for: Impacts of Interleukin-18 Polymorphisms on the Incidence of Delayed-Onset Cytomegalovirus Infection in a Cohort of Kidney Transplant Recipients
Source: Open Forum Infect Dis. 2019 Jul 20;6(9):ofz325. doi: 10.1093/ofid/ofz325 (PMC6798256; doi:10.1093/ofid/ofz325)

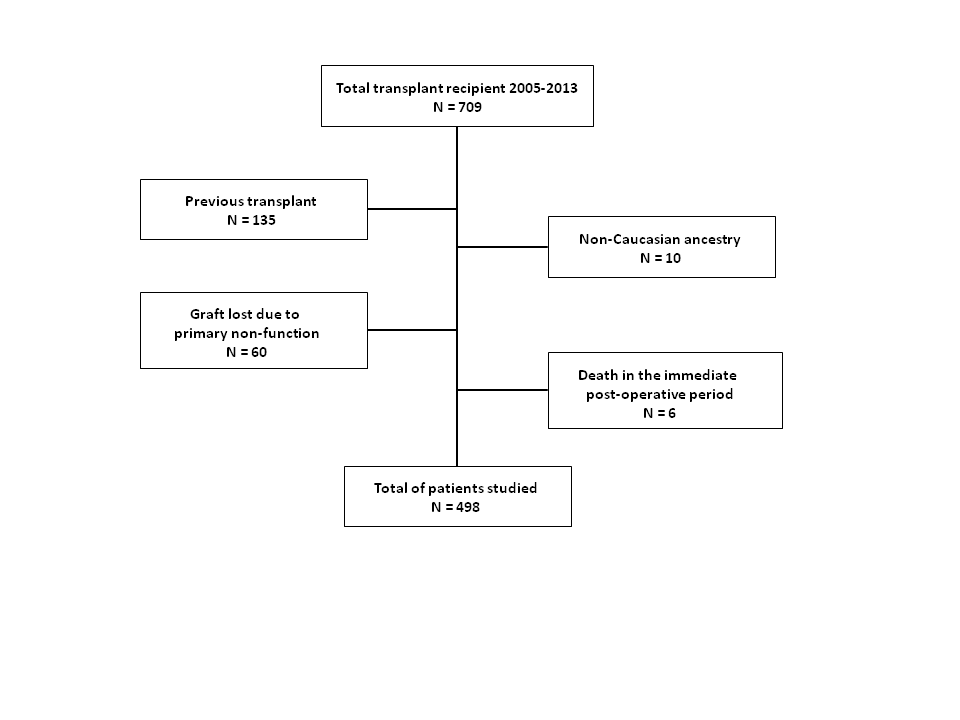

Supplement: ofz325_suppl_supplementary_figure_s1 [file ofz325_suppl_supplementary_figure_s1.png]
